# Spatial Adaptation of Primate Retinal Ganglion Cells Between Artificial and Natural Stimuli

**DOI:** 10.1523/ENEURO.0060-26.2026

**Published:** 2026-04-30

**Authors:** Michaela Vystrčilová, Shashwat Sridhar, Max F. Burg, Mohammad H. Khani, Dimokratis Karamanlis, Helene M. Schreyer, Varsha Ramakrishna, Steffen Krüppel, Sören J. Zapp, Matthias Mietsch, Tim Gollisch, Alexander S. Ecker

**Affiliations:** ^1^University of Göttingen, Institute of Computer Science and Campus Institute Data Science, Göttingen 37037, Germany; ^2^Department of Ophthalmology, University Medical Center Göttingen, Göttingen 37073, Germany; ^3^ Bernstein Center for Computational Neuroscience Göttingen, Göttingen 14195, Germany; ^4^International Max Planck Research School for Intelligent Systems, Tübingen 72076, Germany; ^5^Tübingen AI Center, University of Tübingen, Tübingen 72076, Germany; ^6^International Max Planck Research School for Neurosciences, Göttingen 72074, Germany; ^7^Laboratory Animal Science Unit, German Primate Center, Göttingen 37077, Germany; ^8^ German Center for Cardiovascular Research, Partner Site Göttingen, Göttingen 37073, Germany; ^9^ Cluster of Excellence “Multiscale Bioimaging: from Molecular Machines to Networks of Excitable Cells” (MBExC), University of Göttingen, Göttingen 37075, Germany; ^10^Max Planck Institutefor Dynamics and Self-Organization, Göttingen 37077, Germany

**Keywords:** LN models, receptive fields, retinal ganglion cells, stimulus adaptation

## Abstract

The retina encodes a broad range of stimuli, adapting its computations to features like brightness, contrast, and motion. However, it is unclear whether it also adapts when switching between natural scenes and white noise (WN). To address this, we analyzed the neural activity of male marmoset retinal ganglion cells (RGCs) in response to WN and naturalistic movies. We trained linear–nonlinear models on both stimuli, evaluated their performance, and compared their receptive fields across stimulus domains. We found that models with spatial filters trained on one stimulus ensemble were less accurate when predicting neural activity on the other compared to models trained directly on the target stimulus. This suggests that spatial processing adapts to stimulus statistics. Different RGC types exhibited distinct changes: The OFF midget cells’ receptive fields became enlarged under natural movies (NMs), resulting in a lower cutoff frequency. Parasol cells and large OFF cells did not significantly change their receptive field sizes. All cell types exhibited stronger surrounds under NMs, resembling the whitening filters predicted by efficient coding for stimulus decorrelation, prompting us to test whether these changes were related to the different spectral content of the two stimulus types. Quantifying the effects of the filters’ enhanced surrounds on the stimulus power spectrum showed a significant contribution toward whitening only in ON parasol cells, where a whitening effect emerged regardless of the training stimulus. These results suggest that while RGCs adapt to the differences between WN and NM stimuli, efficient coding can only partially account for this adaptation.

## Significance Statement

Natural scenes differ from artificial stimuli in many properties, including spatial frequency structure. How the retina adapts to these differences remains unclear. To explore this, we studied responses of four primate retinal ganglion cell types to white noise and natural stimuli and compared their receptive field properties. We found that midget cells enlarge their receptive field centers and strengthen their surrounds under natural stimulation, whereas others show enhanced surrounds without center size changes. These modifications qualitatively match predictions of efficient coding based on differences in stimulus power spectra. However, in three of four cell types, stronger surrounds did not substantially whiten responses to natural movies, contrary to theoretical expectations. Thus, efficient coding alone cannot fully account for retinal adaptation mechanisms.

## Introduction

The retina is the direct interface between the visual environment and an animal’s brain. It evolved to encode stimuli with diverse statistical properties by dynamically adjusting to specific attributes of visual inputs. Adaptation to the mean luminance (Dowling, 1960, 1963) and contrast levels (Smirnakis et al., 1997; Kim and Rieke, 2001; Baccus and Meister, 2002) is well documented, while higher-order statistics (skew and kurtosis) showed only mild influence (Tkačik et al., 2014). Salamander and rabbit retinal ganglion cells (RGCs) adapt to spatiotemporal correlations in the stimulus, such as temporal correlations in flickering light intensity, anti-correlated spatial patterns in checkerboard stimuli, and grating orientations (Smirnakis et al., 1997; Hosoya et al., 2005). Adaptation to motion statistics has been shown using moving gratings (Ölveczky et al., 2007) and parameterized motion clouds (Ravello et al., 2019).

Given the many reported forms of adaptation, one might expect neurons to also adapt between a white noise (WN) stimulus and a stimulus with natural statistics. This is indeed the case in later stages of the visual system. For instance, cat V1 simple cells have significantly different spatial receptive fields under WN and natural image stimulation (Sharpee et al., 2006). In the cat’s lateral geniculate nucleus (LGN), Lesica et al. (2007) found that under natural movie (NM) stimulation, the cells’ receptive fields were spatially larger and had a more pronounced surround, compared to WN stimulation. In the retina, Nirenberg et al. (2010) looked at the temporal adaptation of RGCs’ receptive fields between WN and NMs and found different levels of adaptation between ON and OFF cells. However, it is unknown whether the spatial structure of RGCs’ receptive fields adapts to the statistical differences between WN and natural scenes.

Many properties of the visual system have been explained by the efficient coding hypothesis, including the receptive field structure (Atick and Redlich, 1992; Doi et al., 2012; Gupta et al., 2023), the ratio of ON and OFF cells (Ratliff et al., 2010), and the emergence of cell types and their mosaic arrangement (Karklin and Simoncelli, 2011; Ocko et al., 2018; Roy et al., 2021; Jun et al., 2022). Efficient coding posits that neuronal processing minimizes redundant information (Barlow, 1961; Linsker, 1988; Atick and Redlich, 1990).

Lesica et al. (2007) show that LGN neurons adapt their receptive field surround strength and center size in response to WN and NMs in line with what efficient coding would predict given the differences in their spectral frequency power: a stronger surround whitens the correlated movie stimulus, and an increased center size enhances low-pass filtering to remove low-power frequencies in NMs.

Given the optic nerve’s limited capacity, efficient coding could be particularly applicable to the retina. Stimulus adaptation could facilitate more efficient information transmission through this bottleneck. However, it is currently unknown whether the spatial receptive fields of RGCs adjust between WN and NMs. If so, whether this adaptation follows the predictions of efficient coding considering the differences in spectral frequency between the two stimulus types and how this adaptation manifests across cell types. Addressing this gap is crucial for understanding the foundational mechanisms of visual processing and the extent to which the retina contributes to the changes we observe in the LGN.

To answer these questions, we investigate the adaptation in different types of RGCs in the marmoset retina, training linear–nonlinear (LN) models (Marmarelis and Naka, 1972; Korenberg and Hunter, 1986; Sakai et al., 1988; Chichilnisky, 2001) directly on different stimulus types and comparing the resulting model parameters. We show that models perform best when tested on the same stimulus type they were trained on, and performance deteriorates when testing on the other stimulus type, suggesting that RGCs’ receptive fields adapt to stimulus statistics.

Furthermore, we find that midget cells exhibit larger center sizes and stronger receptive field surrounds when responding to naturalistic movies compared to WN. In contrast, parasol and large OFF cells only display stronger surrounds. Although many of these changes are qualitatively in line with the predictions of efficient coding about adaptation to the spatial power spectra of the stimulus types, a quantitative analysis of filtered spectra reveals that the surrounds of OFF midget, OFF parasol, and large OFF cells contribute only minimally to the whitening of the NM signal. In ON parasol cells, we do observe a whitening effect of receptive fields estimated from both WN and NMs. While the whitening in parasol cells can be interpreted as following the efficient coding theory, these findings, together, suggest that additional principles are required to account for retinal adaptation.

## Methods

### Data acquisition and stimuli

We used electrophysiological recordings of the responses of RGCs to artificial and naturalistic stimuli, obtained from adult common marmosets (*Callithrix Jacchus*). The acquisition and preprocessing of this data have been described in detail in Sridhar et al. (2025). In brief, the retina was extracted from the enucleated eye and mounted on a multi-electrode array (MEA) while under constant perfusion with an oxygenated solution and while ensuring minimal light contamination of the tissue. A monochromatic OLED screen, focused on the photoreceptor layer of the mounted tissue, was used to project the stimuli while the activity of RGCs was recorded using the MEA setup. Two types of stimuli were presented: first, spatiotemporal WN was shown to the retinas and after that a naturalistic movie overlaid with simulated eye movements was presented. The resolution of the stimuli was 600 × 800 pixels, the naturalistic movie was shown at full resolution, and the WN was projected at 150 × 200 stimulus pixels, each stimulus pixel corresponding to 4 × 4 screen pixels and each screen pixel to 7.5 × 7.5 μm. Spiking responses of individual RGCs were isolated using a semi-automated spike-sorting pipeline and binned at the resolution of the stimulus refresh rate of 85 Hz for further modeling.

The stimuli were displayed in repeats—trials, containing non-repeating frames which we used for training and validation, and repeating sequences of frames which we used for testing. We used responses of two retinas, with data from one retina being one dataset. Both of the datasets contained 10 trials of spatiotemporal WN stimulus, each consisting of 150 s of non-repeating frames, 30 s of repeating frames, and 20 trials of the naturalistic movie, each consisting of 300 s of non-repeating frames, 60 s of repeating frames.

### Cell selection and classification

The recorded cells were screened on the basis of the reliability of their responses to both stimuli and then assigned cluster labels based on their receptive field and response properties, as described in detail in Sridhar et al. (2025). The cells’ reliability was measured using the fraction of explainable variance (Cadena et al., 2019) and the symmetrized coefficient of determination (Karamanlis and Gollisch, 2021). Four cell clusters were isolated—OFF midget, OFF parasol, ON parasol, and large OFF. While the first three correspond to known cell types in the primate retina, the large OFF cells were composed of a mixture of cell clusters with no known cell type correlates in primates. This left us with 172 reliable, classified cells across two datasets—*Dataset 1* (98 cells) and *Dataset 2* (74 cells).

### LN models

We fit a rank-two, space-time separable difference-of-Gaussians (DoG) LN model to each cell. We refer to this model as the *DoG LN model*. The linear component of the model consisted of two spatiotemporal filter pairs, denoted (*G*_1_, *T*_1_) and (*G*_2_, *T*_2_). The full three-dimensional receptive field *R* was constructed as the difference between the outer products of the spatial and temporal components,

$$R(x,y,t)=G_{1}(x,y)T_{1}(t)-G_{2}(x,y)T_{2}(t).$$

The spatial filters *G*_1_ and *G*_2_ were parameterized as two-dimensional Gaussians whose mean parameters were optimized during model training. To ensure that the resulting spatial profile corresponded to a meaningful DoG, the two Gaussians were constrained to share the same center location and to have the same amplitude sign.

The temporal filters *T*_1_ and *T*_2_ were only constrained to have norm ||*T*|| = 1 such that the amplitudes of *G*_1_ and *G*_2_ would be meaningful but otherwise allowed distinct temporal dynamics for the center and surround components.

The spatial filters had a size of 15 × 15. The temporal filter length was 30 frames, corresponding to a time-window of about 350 ms. To fit each LN model, the stimulus frames were cropped to 15 × 15 pixels around the receptive field center of the cell and then linearly convolved with the spatiotemporal filter pairs before passing through a nonlinearity. The nonlinearity was a softplus function with two learnable parameters, *α* and *β*:

f(x)=αln(1+exp(x+β)).

The receptive field center was taken as the pixel with the largest temporal variance in the spatial receptive field estimate obtained from spike-triggered averaging (STA) on responses to spatiotemporal WN. We calculated the STA as:

$$\text{STA}=\frac{1}{N}\sum_{t=1}^{T}x_{t-\delta \;:\;t}\;y_{t},$$

where *x*_*t*_ is the frame presented at time *t*, *y*_*t*_ is the spike-count recorded at time-point *t*, *δ* is the length of the time-window preceding the spike, *N* is the recorded number of spikes, and *T* is the total number of stimulus frames.

We learned the amplitude, mean, and covariance matrix of the Gaussians *G*_1_ and *G*_2_ for the spatial filters and all values for the temporal filters, along with the parameters of the nonlinearity end-to-end during model training.

### Training

We optimized all parameters of the LN model with stochastic gradient descent without any regularization according to the following training procedure. We shuffled the non-repeating parts of the stimulus trials and assigned 80% of them to the training set and the remaining 20% to the validation set. We randomly initialized the filter values and parameters of the output nonlinearity and updated them by minimizing the Poisson loss:

$$L(r,p)=\sum_{i}^{B}p_{i}-r_{i}\ln\;(p_{i}+\varepsilon ),$$

between the predicted *p*_*i*_ and the observed *r*_*i*_ response values for bin *i*. ε was set to 10^−12^ to avoid computing ln(0). We trained the models for up to 500 epochs, with early stopping when the correlation between the recorded and predicted responses in the validation trials had not improved for 30 consecutive epochs. The optimizer we used was Adam (Kingma and Ba, 2017) with an initial learning rate of 0.005 for NMs and 0.009 for WN. We employed the PyTorch learning rate scheduler ReduceLROnPlateau with a patience of 15 on the validation correlation with a minimal learning rate of 1e^−7^. We used the same number of NM and WN frames to train the respective models.

### Out-of-domain (OOD) adaptation

To adapt the temporal filter and parameters of the nonlinearity obtained from one stimulus to another, we froze the spatial filters. Then, we started training using the same procedure as described above, changing only the parameters of the temporal filters and the nonlinearity. The initial learning rate was 0.001; all the other scheduler settings were kept the same as when training all parameters of the model. We used the same number of NM and WN frames for both training the complete model on one stimulus and adapting the temporal filter and nonlinearity on the other.

### Evaluation

We reported the final performances of the models on the test dataset by averaging the calculated correlation coefficients (CCs) across all cells. For a given RGC *c*, the correlation (Eq. 5) was calculated between the predictions *p*_*c*_ and the trial-averaged firing rates 〈*r*_*c*_〉 on the held-out test sequence with length *T*.

$${\text{CC}}_{c}=\frac{\sum_{t=0}^{T}(\langle r_{c}^{\left(t\right)}\rangle -\langle \bar{r}\rangle )(p_{c}^{\left(t\right)}-\bar{\;p})}{\sqrt{\sum_{t=0}^{T-1}(\langle r_{c}^{\left(t\right)}\rangle -\langle \bar{r}\rangle {)}^{2}}\sqrt{\sum_{t=0}^{T-1}(p_{c}^{\left(t\right)}-\bar{\;p}{)}^{2}}},$$

where $\bar{\;p}$ is the within trial-average prediction and $\bar{r}$ the within trial-averaged response.

### Receptive field size and surround amplitude estimation

We used the parameters of the DoG LN models to estimate the size of the cells’ receptive fields and the strength of the surrounds. We defined the receptive field size *S* as

$$S=4\pi \sqrt{\lambda_{1}\lambda_{2}},$$

where *λ*_1_ and *λ*_2_ are the eigenvalues of the covariance matrix of the *G*_1_ spatial Gaussian.

The surround amplitude was estimated from the full three-dimensional receptive field estimate *R*. First, we identified the *max frame*, defined as the temporal frame in which the pixel value at the learned center-coordinate was maximal for ON cells or minimal for OFF cells. Because the surround component may precede the center in time (Kilavik et al., 2003; Cowan et al., 2016), estimating surround strength directly from the max frame may underestimate its amplitude. Furthermore, using the relative amplitudes of *G*_1_ and *G*_2_ alone may yield imprecise estimates due to interactions between their spatial scale and the unconstrained temporal filters.

To address these issues, we identified a *surround frame* (Fig. 3*B*). The surround frame was selected from a temporal window of ±6 frames around the max frame, under the constraint that the pixel value at the center-coordinate retained the sign of the cell’s polarity (positive for ON cells, negative for OFF cells). Among these frames, the surround frame was defined as the frame containing the pixel with the largest absolute value of the opposite polarity (negative for ON cells, positive for OFF cells). The largest-magnitude pixel value of opposite polarity in the surround frame was taken as the surround amplitude.

### Mean receptive field estimation

We normalized the LN model filters such that the maximum of the temporal filter was set to 1 (−1) for ON (OFF) cells and averaged across the *surround frames* of the cells to calculate the mean receptive field for each cell type. Before calculating the average, we aligned the centers of all spatial filters within a cell type using the center coordinates from their DoG fits. Empty pixels created due to the center shift were replaced by mean gray.

### Alternative LN model

To validate the robustness of our results, we trained an additional space-time separable rank-one LN model consisting of a single spatial and temporal filter pair. The spatial filter was cropped to 15 × 15 pixels and all 225 parameters were learned directly end-to-end, without imposing a Gaussian parameterization. The temporal filter length was 30 frames, matching the DoG LN model.

### Alternative LN model receptive field size and surround amplitude estimation

Using the alternative rank-one LN model, we estimated receptive field center size and surround strength using two independent methods, described below.

1) Post hoc DoG fitting of rank-one spatial filters.

For the rank-one LN model, center size and surround amplitude were first estimated by fitting a DoG model to the learned spatial filter post hoc. The DoG was defined as

$$\mathrm{DoG}(x)=A_{c}\exp\;\left(-\frac{1}{2}(x-\mu {)}^{T}{\mathrm{cov}}_{c}^{-1}(x-\mu )\right)-A_{s}\exp\;\left(-\frac{1}{2}(x-\mu {)}^{T}{\mathrm{cov}}_{s}^{-1}(x-\mu )\right),$$

where *A*_c_(*A*_s_) is the center (surround) amplitude, *μ* the position vector shared by both center and surround and ${\mathrm{cov}}_{c}=\left(\begin{array}{cc} \sigma_{cx}^{2} & \sigma_{cxy}\\ \sigma_{cxy} & \sigma_{cy}^{2} \end{array}\right),{\mathrm{cov}}_{s}=\left(\begin{array}{cc} \sigma_{sx}^{2} & \sigma_{sxy}\\ \sigma_{sxy} & \sigma_{sy}^{2} \end{array}\right)$ are the covariance matrices of the center (surround) Gaussians in pixel space. The DoG was fitted by minimizing the mean squared error between the DoG estimate and the spatial filter. We applied a positivity constraint on the diagonal elements of the covariance matrices of both Gaussians and enforced that both have the same amplitude sign. The initial parameters were *A*_c_ = 0.5, *σ*_c*x*_ = 1.2, *σ*_c*y*_ = 1.2, *σ*_c*xy*_ = 10^−3^, *A*_s_ = 0.5, *σ*_s*x*_ = 1.5, *σ*_s*y*_ = 1.5, *σ*_s*xy*_ = 10^−3^ and the mean *μ* was set as the center of the crop region.

We established the center size *S* as $S=4\pi \sqrt{\lambda_{1}\lambda_{2}}$, where the *λ*_1_ and *λ*_2_ were the eigenvalues of the center covariance matrix. To obtain the surround strength, the DoG-parameterized spatial filter was normalized to a maximum absolute value of 1. Then, the absolute value of the lowest negative value (if the center amplitude was positive) or the highest positive value (if the center amplitude was negative) of the DoG fit was taken to be a measure of the surround strength.

2) Smoothing- and threshold-based estimation

As a complementary, nonparametric approach, we also estimated center size and surround amplitude directly from the learned spatial filters using smoothing and thresholding. In this method, the filters were pre-processed with a Kaiser window (*β* = 7) to reduce edge artifacts. The filter was then normalized by its maximal absolute value. The center region was identified by locating the pixel corresponding to the peak maximum for ON cells or minimum for OFF cells. A binary mask was created by thresholding at 20% of the peak value, and the connected component containing the peak was isolated. The area of this central region was the resulting size of the receptive field center. To estimate the surround amplitude when using the thresholding method, the windowed, normalized LN filter was first smoothed with a Gaussian kernel (*σ* = 1.5). After that, the maximum (for OFF-type filters) or minimum (for ON-type filters) of the smoothed filter was taken as the surround response amplitude.

### Simulated filters

We simulated spatial filters inspired by the principles of efficient coding. First, we simulated filters mimicking those obtained from WN data, i.e., filters with a small center size and no surround. We call these the “white noise” filters. To generate them, we used parameters of the central Gaussian of a DoG fit to the mean midget cell receptive field, with amplitude normalized to 1, and constructed a diagonal covariance matrix with all diagonal elements equal to the largest diagonal value in the covariance matrix of the DoG fit. This resulted in a circular receptive field of the size of a midget cell with no surround. We followed the same procedure with the mean parasol cell receptive field to simulate large center sizes and produce stronger low-pass filtering. We call these the “low-pass” filters.

To simulate the presence of a surround in both the “white noise” and the “low-pass” filters, we subtracted a second 2D Gaussian from each. The amplitude and covariance matrix of the second Gaussian were adjusted manually so as to ensure the resulting DoG filter would lead to a flattening of the low-frequency spatial power spectrum for naturalistic movies (see below).

### Power spectra calculation

We calculated the spatial power spectral densities of non-filtered WN and NM stimuli, both at monitor pixel resolution, by performing a 2D Fast Fourier Transform (FFT) on each 800 × 600 stimulus frame cropped to 600 × 600 pixels. Radial averaging was applied to the frequency components of each frame, and the results were averaged across frames to obtain the final stimulus power spectra.

To get transfer functions of the learned filters, we padded each filter to match the 600 × 600 stimulus dimensions. We used the same FFT-based procedure, computing individual transfer functions for each LN model’s spatial filter. Cell-type-specific transfer functions were obtained by averaging across filters from the same cell type. Transfer functions for the simulated filters were computed in the same way as for the learned filters.

We calculated the spectrum of the NM stimulus filtered with both rank-one and rank-two estimated receptive fields from both WN and NMs. We followed the same procedure in all the settings. We first convolved the NM stimulus with each cell’s estimated receptive field. We then computed the spatial power spectra of the filtered stimulus frames. To get the spectrum for a given cell type, we averaged over the spectra filtered by cells classified as such. For the simulated filters, we multiplied their transfer functions with the power spectra of each stimulus frame and then averaged over the filtered frames.

## Results

**Figure 1.**
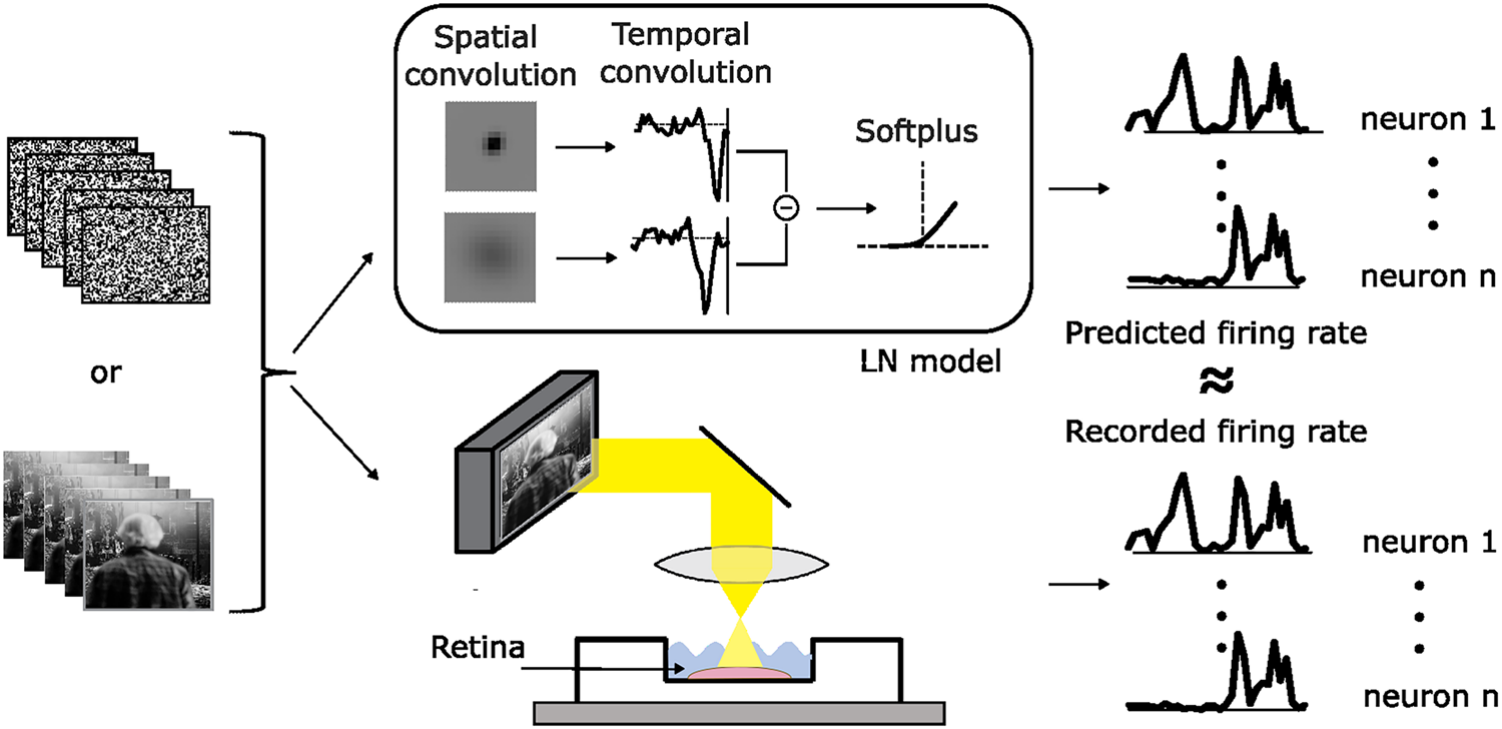
We presented white noise or natural movie stimuli to a retina while recording ganglion cell responses using a micro-electrode array. We train end-to-end LN models to predict the average firing rate of all neurons given the stimulus in a time window preceding the prediction.

We analyzed the activity of marmoset RGCs in response to WN and NM visual stimuli recorded using MEAs (Fig. 1). We will also refer to these stimuli simply as “noise” and “movies.” Our datasets contained the activity of 172 RGCs across two marmoset retinas from Sridhar et al. (2025), which were shown both movie and noise stimuli. Sridhar et al. (2025) classified these cells into four groups—OFF Midget, OFF Parasol, ON Parasol, and Large OFF—which we also used, and excluded cells that responded unreliably (for further details, see Methods). The large OFF group was an inhomogeneous class of neurons that contained multiple cell clusters with spatially large receptive fields and consistent response characteristics but without known cell type correlates in primates.

We split the stimuli into non-repeating, unique video sequences for training models (“training set”) and a smaller number of repeated video segments for evaluating the final model performance (“test set”). A sequence of a non-repeating segment followed by a repeated segment comprised a “trial.” In our data, we showed 10–20 stimulus trials to the retina. The length of a non-repeating segment was 150–300 s and of the repeating segment 30–60 s, depending on the stimulus type.

We trained LN models to predict the neural responses in these datasets (Fig. 1). To evaluate their performance, we computed the CC between the predicted spike counts and the trial-averaged neuronal response, which we measured in 1/85-s time bins (corresponding to the frame rate of 85 Hz) on the test segments (Eq. 5). We held out the averaged test segments during training and trained the models only on non-repeating training set data.

### Training stimulus-specific models improves predictive performance

LN models are a classical way of modeling RGC responses to WN. We started by establishing their predictive performance on both WN and NM stimuli to verify that they are reliable models of the studied RGCs.

Our LN models consisted of two space-time separable linear filter pairs, denoted (*G*_1_, *T*_1_) and (*G*_2_, *T*_2_), followed by a static nonlinearity. The spatial components *G*_1_ and *G*_2_ were parameterized as two-dimensional Gaussians representing the center and surround components of the receptive field, respectively. The temporal components *T*_1_ and *T*_2_ were normalized during training but otherwise unconstrained. The full spatiotemporal filter was given by the difference between the outer products *G*_1_ *T*_1_ − *G*_2_ *T*_2_. Predicted spiking responses were obtained by taking the inner product between the stimulus and the spatiotemporal filter and passing the result through a parametrized softplus nonlinearity (Fig. 1).

**Figure 2.**
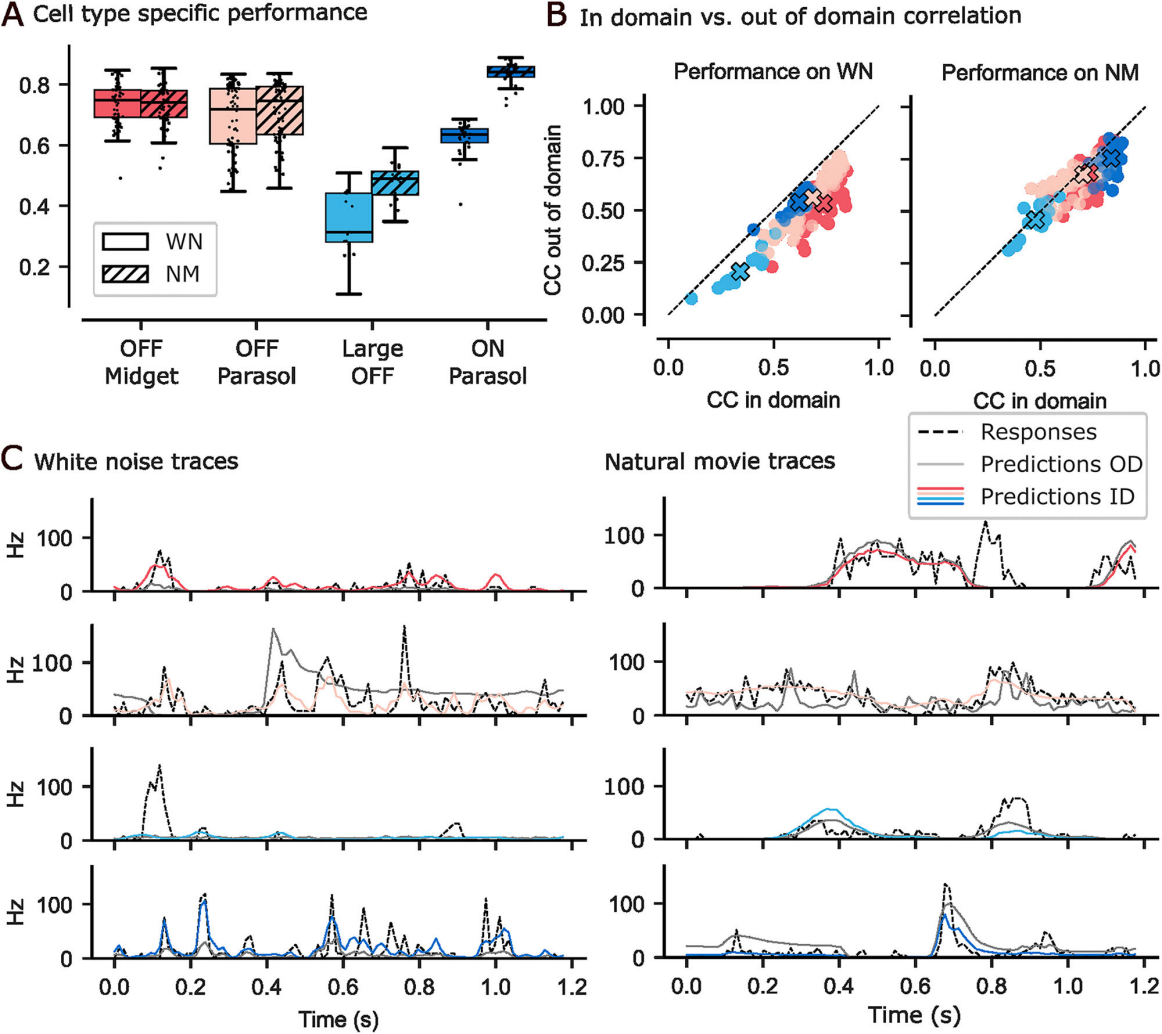
***A***, LN model correlation coefficient (CC) for the different cell types on white noise (WN; solid) and natural movies (NM; dashed), respectively. ***B***, In-domain (ID) versus out-of-domain (OOD) correlation comparison. Left plot: *x*-axis are models trained and evaluated on WN, *y*-axis are models trained on NMs and adapted and evaluated on WN. Right plot: *x*-axis are models trained and evaluated on NMs, *y*-axis are models trained on WN and adapted and evaluated on NMs. ***C***, Comparison of example cells’ prediction for each cell type between the recorded signal (black, dashed), ID predictions (colored), and OOD predictions (gray).

We obtained the filters and nonlinearity parameters of the LN models by fitting them directly on the training set of the data using maximum likelihood estimation with stochastic gradient descent. We fit models independently for each stimulus type—that is, two models per cell, one for WN and one for NMs. First, we evaluated the models *in-domain* (ID), i.e., the models trained on WN data were evaluated on the WN test data split, and models trained on NMs were evaluated on the NMs test data split (Fig. 2*A*). The Pearson’s CC between the recorded and model-predicted RGC activity, mean and standard deviation (SD) across cells of a given cell type, were between 0.34 (±0.1 SD) for large OFF cells and 0.74 (±0.07 SD) for OFF midget cells (OFF parasol: 0.69 ± 0.1 SD; ON parasol: 0.62 ± 0.05 SD).

On NMs, the models performed better for all four cell types, with the mean correlation between 0.48 (±0.07 SD) for large OFF cells and 0.83 (± 0.02 SD) for ON parasol cells (OFF midget: 0.73 ± 0.06 SD; OFF parasol: 0.71 ± 0.09 SD).

Using correlation as a metric to compare stimulus conditions may be problematic, because naturalistic stimuli could evoke neuronal responses with different amplitude and sparsity characteristics than WN. To determine whether such differences could systematically bias correlation values, we quantified each cell’s intrinsic trial-to-trial reliability by computing correlations between averaged odd and even trials for both stimulus types. The average correlation across cells was 0.77 ± 0.07 (SD) for WN and 0.77 ± 0.10 for NMs, suggesting that differences in response reliability are not a causal factor.

Next, we asked whether models trained on one stimulus would generalize to the other stimulus—that is, we evaluated their performance *OOD*. Specifically, we were interested in whether the spatial part of the receptive field is stimulus-specific or generalizes OOD, i.e., to the other stimulus. Hence, we took a model trained on one stimulus type (for example, WN) and froze its spatial filter weights. We then continued training this model on the other stimulus (NMs in this example), adjusting only the parameters of the temporal filter and the nonlinearity. Finally, we evaluated the model on the test set of the other stimulus (NMs). This approach allowed us to determine if the spatial filter generalized across stimuli. If the OOD model’s performance matched the ID performance, it would indicate that the spatial filter is a fixed property of the neuron. If not, it would suggest that the spatial receptive field adapts, as only the spatial filter was held constant across evaluations.

We found that all models performed better in an ID evaluation setting compared to OOD evaluation (Fig. 2*B*). The margin in correlation of the ID over the OOD model on WN was between 0.2 for OFF midget cells and 0.08 for ON parasol cells (OFF parasol: 0.12; large OFF: 0.14). On NMs, this difference was 0.02 for large OFF cells, 0.03 for OFF parasols, 0.05 for OFF midgets, and 0.08 for ON parasol cells. This means that the spatial receptive fields of our LN models did not generalize across stimuli, suggesting spatial changes in retinal computation between these two stimuli. The traces of example cells for the different cell types also show that the ID models were able to better follow the recorded firing rates compared to OOD models (Fig. 2*C*).

The WN models generalized better to natural stimuli than the other way around. An alternative possible explanation to that of the RGC receptive fields adapting, stemming from this observation, is that the NM stimulus does not constrain the model as effectively, leading to poorer OOD performance. However, if the WN-trained filters were more accurate representations of the RGCs receptive fields, we would expect them to also include a surround—a known feature of RGCs. Moreover, even though the differences between ID and OOD models evaluated on NMs were minor, this does not imply small changes in the receptive fields between the two stimuli. The NM stimuli have most of their energy in the low-frequency range, which means that a major fraction of the correlation was explained by a few low-frequency components, whereas, as we show below, the receptive field differences were larger in higher frequencies.

### RGCs adapt their spatial receptive fields to stimulus statistics

**Figure 3.**
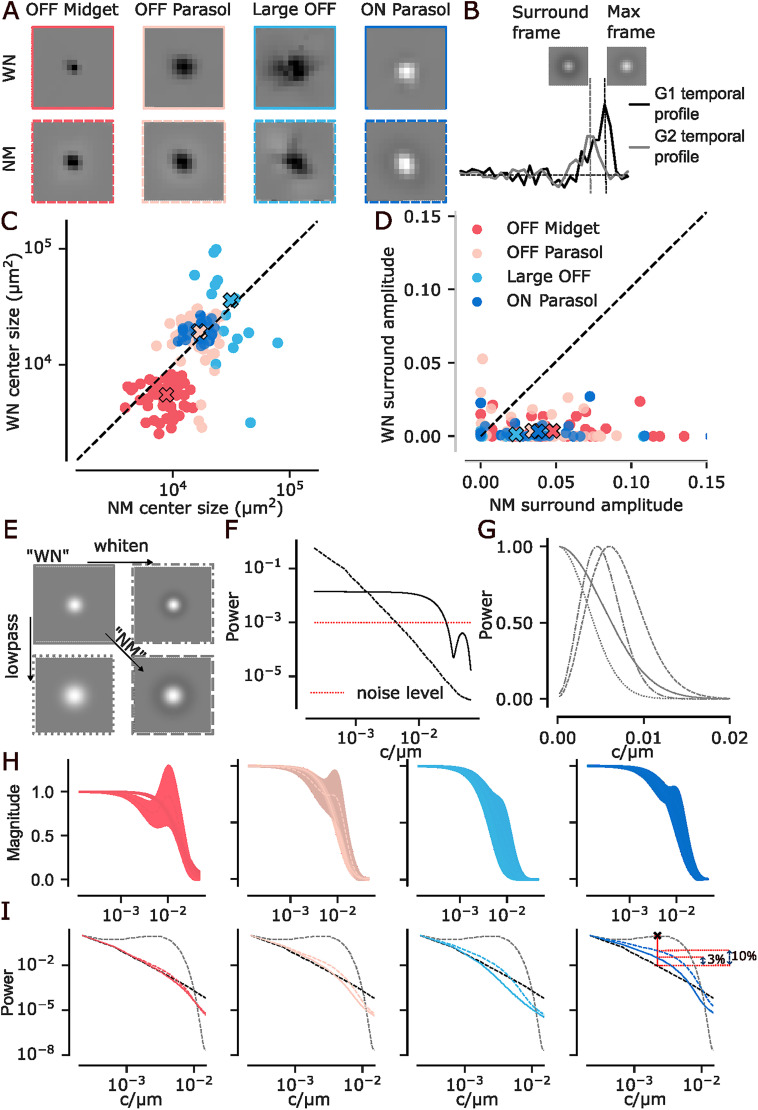
***A***, Cell-type-specific spatial receptive field comparison: Average spatial receptive field across neurons. First row: white noise (WN). Second row: natural movies (NMs). ***B***, Illustration showing how the surround frame is selected to estimate the surround amplitude. The max frame is defined as the temporal frame with the maximal (ON cells) or minimal (OFF cells) center-coordinate filter value, corresponding to the largest difference between the G1 (black) and G2 (gray) temporal profiles. The surround frame is then selected from a temporal window around the max frame as the frame in which the receptive field contains the largest absolute value of opposite polarity, while the center pixel has the same polarity as in the max frame. ***C***, Size of receptive field center for WN versus NMs. ***D***, Strength of receptive field surround for white noise versus NMs. Values below 1e^−4^ clipped to 0. ***E***, Illustration of how the size of the receptive field center and the magnitude of the surround affect filter properties. Horizontal: surround contributes to whitening. Vertical: increased size contributes to low-pass filtering. The combination (bottom right) would be the prediction of efficient coding. ***F***, Power spectrum of NM (black, dashed) and white noise stimulus (black, solid). Red dotted line: Flat spectrum of a hypothetical noise level. ***G***, Transfer functions of the four theoretical filters from ***E***. Line styles correspond to the outlines of the boxes in ***D***. ***H***, Average transfer functions of the cell-type-specific receptive fields from LN models trained on white noise (solid line) and NMs (dashed) for OFF midget (first plot, red), OFF parasol (second plot, pink), large OFF (third plot, turquoise), and ON parasol cells (fourth plot, blue). Shaded regions: SD. ***I***, Power spectrum of the original NM (black, dashed) with NM spectra after filtering with different filters: the simulated whitening filter (gray, dashed; panel ***E***, bottom right), the rank-one model receptive fields estimated from movies (light colored dashed lines) and white noise (light colored solid lines) and the rank-two model receptive fields estimated on movies (dark colored dashed lines) and white noise (dark colored solid lines) for different cell types. The red vertical line represents the distance between the original NM spectrum and a flat spectrum at the receptive field relevant frequency. WN, rank-two estimated receptive field flattens it by 3% (distance between the first two dotted red lines), NM rank-two estimated receptive field flattens it by 10% (distance between the first and third dotted red lines). All spectra are normalized to the first data point.

The lack of generalization across the WN and NM stimuli suggests that some features of the receptive fields depend on the type of stimulus. We isolated the generalization of the spatial component of the receptive field and examined its adaptation in size and surround strength. First, we visualized the average receptive field for each cell type by aligning all receptive fields to the same location and averaging across neurons of the same type (Fig. 3*A*). Spatial receptive fields differed qualitatively across cell types. For midget cells, the center receptive field size increased under NM stimulation compared to WN. For ON and OFF parasol cells and large OFF cells, the center size stayed roughly unaffected. While the changes in size varied across cell types, we observed a more pronounced surround under NMs compared to WN for all cell types.

To corroborate these qualitative results, we quantified the center size and surround amplitude from the spatial Gaussians *G*_1_ and *G*_2_ and from the full three-dimensional spatiotemporal receptive field *R*. The center size was calculated as the area encompassing two standard deviations of *G*_1_. The surround amplitude was defined as the absolute value of the minimum (maximum) of the full three-dimensional filter for ON (OFF) cells, respectively, evaluated at the *surround frame* (Fig. 3*B*). The surround frame was the temporal frame, selected from a window around the frame with the maximal (ON cells) or minimal (OFF cells) center-coordinate filter value, in which the center-coordinate filter value retained the sign of the cell’s polarity and in which the receptive field contained the largest absolute value of the opposite polarity.

This quantitative analysis showed a significant increase in size for midget cells (Wilcoxon signed-rank test, *p* < 0.001, *N* = 51) and a significant increase in surround amplitude strength for all cell types (individual tests for cell types, Wilcoxon signed-rank test, OFF midget: *p* < 0.001, *N* = 51; OFF parasols: *p* < 0.001, *N* = 69; large OFF *p* = 0.006, *N* = 16; ON parasol: *p* = 0.001, *N* = 36; Fig. 3*C,D*).

Because the Gaussian fits to the spatial filters might be too rigid, we trained an additional space-time separable rank-one LN model consisting of a single spatial and temporal filter pair. The spatial filter was cropped to 15 × 15 pixels and all 225 parameters were learned directly end-to-end, without imposing a Gaussian parameterization. The temporal filter length was 30 frames, matching the DoG LN model.

Using this LN model, we estimated receptive field center size and surround strength using two independent methods. When analyzing the spatial filter using these two methods, we found the same effects as when fitting our DoG LN model, both quantitatively (Fig. 1-1*A*) and qualitatively. Midget cell size increased under NM simulation, and parasol cell sizes stayed roughly the same. In the case of the large OFF cells, they decreased in size when using this method, as opposed to also staying roughly the same when estimated from the parameters of the DoG LN model. We attribute this difference to the large OFF cells in the dataset being the least reliable and least well predicted, suggesting their receptive field estimates are also the noisiest (Fig. 1-1*B,D*). The increase in surround strength under NMs, compared to WN, for all cell types was identified also using this method (Fig. 1-1*C,E*).

10.1523/ENEURO.0060-26.2026.f1-1 Figure 1-1 **A.** Cell-type-specific spatial receptive field comparison: Average spatial receptive field across neurons. First row: white noise. Second row: natural movies. **B., D.** Size of receptive field center for white noise vs. natural movies when fitting a DoG to the spatial filter post-hoc (B) and when using smoothing and thresholding (D). **C., E** Strength of receptive field surround for white noise vs. natural movies when fitting a DoG to the spatial filter post-hoc (C), and when using smoothing and thresholding (E). Values below 1*e*^−4^ clipped to 0. Download Figure 1-1, TIF file.

### Surrounds contribute little to spatial whitening of the responses to natural stimuli

We asked what could explain these receptive field changes. They resembled changes predicted by efficient coding (Fig. 3*A*). Based on the efficient coding hypothesis (Atick and Redlich, 1992), we can expect two ways in which the receptive fields should adjust between WN and NM stimulation if the information about the stimulus being transmitted from the eye to the brain is to be maximized, considering constrained resources. First, the filter for NMs should have a center-surround structure to remove correlations that are present in natural environments. The noise stimulus is already white in the frequencies influenced by RGCs’ receptive fields, and, therefore, the receptive fields should not show a surround (Fig. 3*D*, horizontal).

Second, the receptive fields should be larger for NM stimuli compared to WN stimuli because of the difference in power in high frequencies and the resulting signal-to-noise ratio (SNR) of the two stimuli (Fig. 3*D*, vertical). NMs exhibit a 1/*f*^2^ spectrum, where the power decreases steeply with frequency. Therefore, the SNR of the movie stimulus also drops faster with increasing frequencies, compared to the almost flat spectrum of the WN stimulus. To illustrate this, we consider photon noise with a flat spectrum. When a stimulus power spectrum intersects with the photon noise spectrum, the SNR becomes too low for meaningful information extraction. For the NM spectrum, this intersection occurs at a lower frequency compared to the flat spectrum of the WN stimulus (Fig. 3*E*). As a result, the high-frequency information becomes too unreliable in NMs, resulting in larger filters with lower cutoff frequencies being optimal (Fig. 3*F*, dashed). Conversely, WN retains a sufficiently high SNR at higher frequencies, allowing for smaller filters with higher cutoff frequencies, therefore including higher-frequency information (Fig. 3*F*, solid).

Midget cells were the only cell type where the predicted increase in the size of the center occurred when comparing NM stimulation to WN stimulation. The increase in surround strength under NM stimulation occurred for all cell types. We previously quantified the increase in the strength of the surround (Fig. 3*C*). However, the question remains how much this increase in surround contributes to the whitening effect predicted by efficient coding. To quantify this, we filtered the NM signal using the LN model-estimated receptive field filters. Then, we computed the spatial power spectra of the filtered signal and averaged them over the cells of each cell type.

The whitening effect was negligible for all cell types except for ON parasol cells. For ON parasol cells, filters estimated from WN indeed flattened the spectrum somewhat (by 3%) relative to a flat spectrum. Those estimated from NMs achieved a stronger (10%) flattening (Fig. 3*H*). In contrast, a flattening of 88% was achieved by synthetic filters, which we optimized to whiten the movie signal, using the same receptive field sizes as for the RGCs (Fig. 3*H*). These percentages are reported for the spatial frequency at which the whitening effect was maximal within the relevant range, given the receptive field sizes. Thus, ON parasol cells did whiten the spectra to some extent under both stimuli, more so under NMs, qualitatively following the efficient coding hypothesis at least to some extent. On the other hand, three out of four cell types we studied did not display an increase in whitening despite exhibiting a stronger surround under NM stimulation. This result implies that the surround serves a purpose different from that of efficient coding.

## Discussion

### LN models

In this paper, we demonstrate that LN models yield distinct response functions, depending on the stimulus ensemble on which they are trained: either WN or natural scenes. Several previous studies have advocated for using natural scenes to study visual processing, as models trained on WN stimuli do not generalize well to NM inputs (Heitman et al., 2016; McIntosh et al., 2017; Sinz et al., 2018). Our results differ from these earlier findings and extend them in several ways:

First, also in our hands, LN models trained on noise perform worse on movies compared to models directly trained on movies. However, unlike reported by Heitman et al. (2016), we find this performance gap to be rather small. Second, we also observed the converse effect: Training models only on movies does not allow them to predict how RGCs respond to noise accurately. This finding contrasts with work in the primary visual cortex, where generalization from movies to noise was better than from noise to movies (Talebi and Baker, 2012; Sinz et al., 2018). However, we note that care is needed when interpreting such numbers. Overall, responses to movies were better predicted in our data than responses to noise, presumably because movies contain stronger local luminance fluctuations, which are “easy” to predict and account for a larger fraction of the total variance compared to noise stimuli. Thus, interpreting absolute differences in predictive performance may be challenging. Generally, performance metrics based on explained variance are somewhat challenging to interpret. While only a perfect model would achieve 100% performance, differences across models do not necessarily indicate how interesting the explained variance is in terms of the underlying computational phenomena it captures.

Another possible explanation for the asymmetry in generalization is that the NM stimulus does not constrain the model as tightly as WN, leading to poorer parameter estimates and reduced performance when NM models are applied to WN.

However, we do not believe this fully explains the results. If the WN-trained filters were indeed more accurate representations of the cell’s computation, we would expect them to include a surround, which is a well-established feature of RGCs. Their absence suggests that the cell itself does not engage the surround under WN stimulation, rather than the model failing to recover it.

### Spatial adaptation

Our findings demonstrate that RGCs adapt their spatial receptive fields in response to differences between spatiotemporal WN and NM stimuli. While ideally, one would estimate receptive field properties using a third, shared probe stimulus to help isolate true functional changes from differences in how well receptive fields can be measured under each stimulus, such data were not available in our case, so we assessed both adaptation and receptive field structure using the same stimulus ensemble.

We observed two types of changes—in the receptive field center sizes and surround amplitude strength. The observed receptive field size changes varied across cell types. OFF midget cells showed increased receptive field center sizes under NM stimulation. This increase is consistent with the expected adaptation to the lower SNR at high frequencies in NMs compared to WN, as can be derived from the efficient coding theory (Atick and Redlich, 1992). Parasol and large OFF cells exhibited no significant size changes, potentially because their receptive fields are already large enough to function as low-pass filters effectively. The increase in surround amplitude strength occurred for all cell types.

Similar changes have been reported in higher visual areas. Lesica et al. (2007) also found that in the cat LGN, the cells’ receptive fields increased both the center size and surround amplitude strength under natural stimuli compared to WN stimulation. Given the resemblance of the adaptation we observed in the RGCs, specifically the OFF midget cells, it could originate in the retina. Sharpee et al. (2006) demonstrated that receptive fields of cat V1 neurons adapt to stimulus statistics by tuning toward lower spatial frequencies under NM stimulation compared to WN. This tuning aligns with the SNR adaptation predictions and matches the receptive field changes of midget cells observed in our study.

While natural scenes and WN differ along many dimensions beyond spatial frequency, such as higher-order statistics, we focused our analysis on spatial frequency because the changes we observed—such as increased surround strength and altered receptive field sizes—qualitatively resemble predictions from the efficient coding theory and the adaptation to higher order statistics is limited in the salamander retina (Tkačik et al., 2014). This motivated us to ask whether these changes serve to process the input in a way that reduces spatial redundancy, as efficient coding would predict for stimuli with differing spatial frequency content.

### The efficient coding theory

There are two key aspects to consider regarding efficient coding theory in light of the receptive field structure and changes we show: (1) its general prediction about receptive field structure, such as the center-surround organization, and (2) its prediction about receptive field adaptation to specific stimulus statistics, such as stronger surrounds under NM stimulation.

The observed center-surround structure in ON parasol cells, despite its low strength, could support the idea that these cells have evolved to process the general statistics of the visual environment, in line with efficient coding. Their receptive fields may be sub-optimal for WN but are well-suited to partly decorrelate natural scenes by whitening the signal (Atick and Redlich, 1992). However, the extent of whitening in the ON parasol cells—13% (5%) when filtering with movie (noise) estimated filters relative to a fully whitened, i.e., flat spectrum—and the absence of any significant whitening in the other three cell types suggests that the efficient coding principle may not fully explain their receptive field structure.

More broadly, characterizing neural properties—such as receptive field features—and comparing them to predictions of existing theoretical frameworks is essential for advancing our understanding of neural computation. Such descriptions reveal where current frameworks capture important aspects of neural processing and where they fall short. Moreover, they provide concrete empirical constraints that can guide the development of future theories capable of explaining these observations.

The receptive field surround is not the only mechanism by which the retina could achieve whitening of natural stimuli. Multiple studies have suggested other decorrelation mechanisms, such as a nonlinear transformation at the output stage of the retina (Pitkow and Meister, 2012; Turner et al., 2018) and fixational eye movements (Kuang et al., 2012; Segal et al., 2015). Therefore, it is still possible that the retina whitens the input signal for efficient encoding. For this reason, we do not invalidate the efficient coding theory as a whole; our contribution lies in establishing that the presence of a surround in the receptive field does not automatically translate to whitening.

A linear receptive field that whitens also does not necessarily directly translate to decorrelated cell responses. It is also important to consider the nonlinearity of the receptive field itself. While classical LN models consider the center and surround as additive components, recent studies have shown that the interplay between both components could also be multiplicative or involve more complex nonlinearities (Turner and Rieke, 2016), and these nonlinearities can evoke correlated activity (Karamanlis et al., 2025).

Regarding receptive field adaptation across stimulus statistics, the midget cells did increase in size as would be predicted by efficient coding. And while the other cell types did not, it could be, as stated above, due to their receptive field already being large enough to be effective low-pass filters. Concerning the surround, only ON parasol cells demonstrated a stronger surround that could contribute to whitening under NMs compared to WN, consistent with the efficient coding theory predicting stimulus-specific encoding. Yet, the WN filter for ON parasol cells produced a more substantial whitening effect than the NM filters of all other cell types. This observation suggests that it is not necessarily the change in stimulus that is responsible for the center-surround receptive field structure. Rather, it appears that ON parasol cells have a more whitening receptive field compared to the other cell types, independent of stimulus statistics.

### Temporal adaptation

Nirenberg et al. (2010) focused on temporal adaptation between WN and NM stimuli of RGCs in the retina. We did not study temporal adaptation for two reasons. Firstly, we pool multiple retinas, and the temporal profiles of cells of the same cell types are not well comparable across retinas (Zhao et al., 2019). Secondly, the NM stimulus was presented at a 25% lower mean luminance than the WN stimulus, which could additionally affect the temporal properties of the cells (Benardete and Kaplan, 1999).

While the changes in luminance also affect the spatial properties of RGCs’ receptive fields, they cannot account for the adaptation we observed. Lower luminance tends to increase the size of the receptive field, but this change generally occurs at a threshold when switching from predominantly cone to predominantly rod vision (Troy et al., 1999). Neither of our stimuli is dark enough to trigger this switch. Lower luminance also decreases the strength of the surround (Thoreson and Mangel, 2012; Farrow et al., 2013). In our case, however, the stimulus with the higher surround strength is the one with a lower luminance. Therefore, it is safe to assume that the surround that appears does not do so because of a luminance decrease but rather due to differences in stimulus statistics.

### Data and code availability

The data is available on GIN and the code on GitHub

## Synthesis

Reviewing Editor: David Schoppik, New York University Grossman School of Medicine Decisions are customarily a result of the Reviewing Editor and the peer reviewers coming together and discussing their recommendations until a consensus is reached. When revisions are invited, a fact-based synthesis statement explaining their decision and outlining what is needed to prepare a revision will be listed below. The following reviewer(s) agreed to reveal their identity: NONE. Note: If this manuscript was transferred from JNeurosci and a decision was made to accept the manuscript without peer review, a synthesis may not be available.

To the extent possible, the revised manuscript addresses the reviewers' concerns and is stronger for the clarifications.
