# Supplementary figures and images for: Spatial Adaptation of Primate Retinal Ganglion Cells Between Artificial and Natural Stimuli
Source: eNeuro. 2026 Apr 30;13(5):ENEURO.0060-26.2026. doi: 10.1523/ENEURO.0060-26.2026 (PMC13138850; doi:10.1523/ENEURO.0060-26.2026)

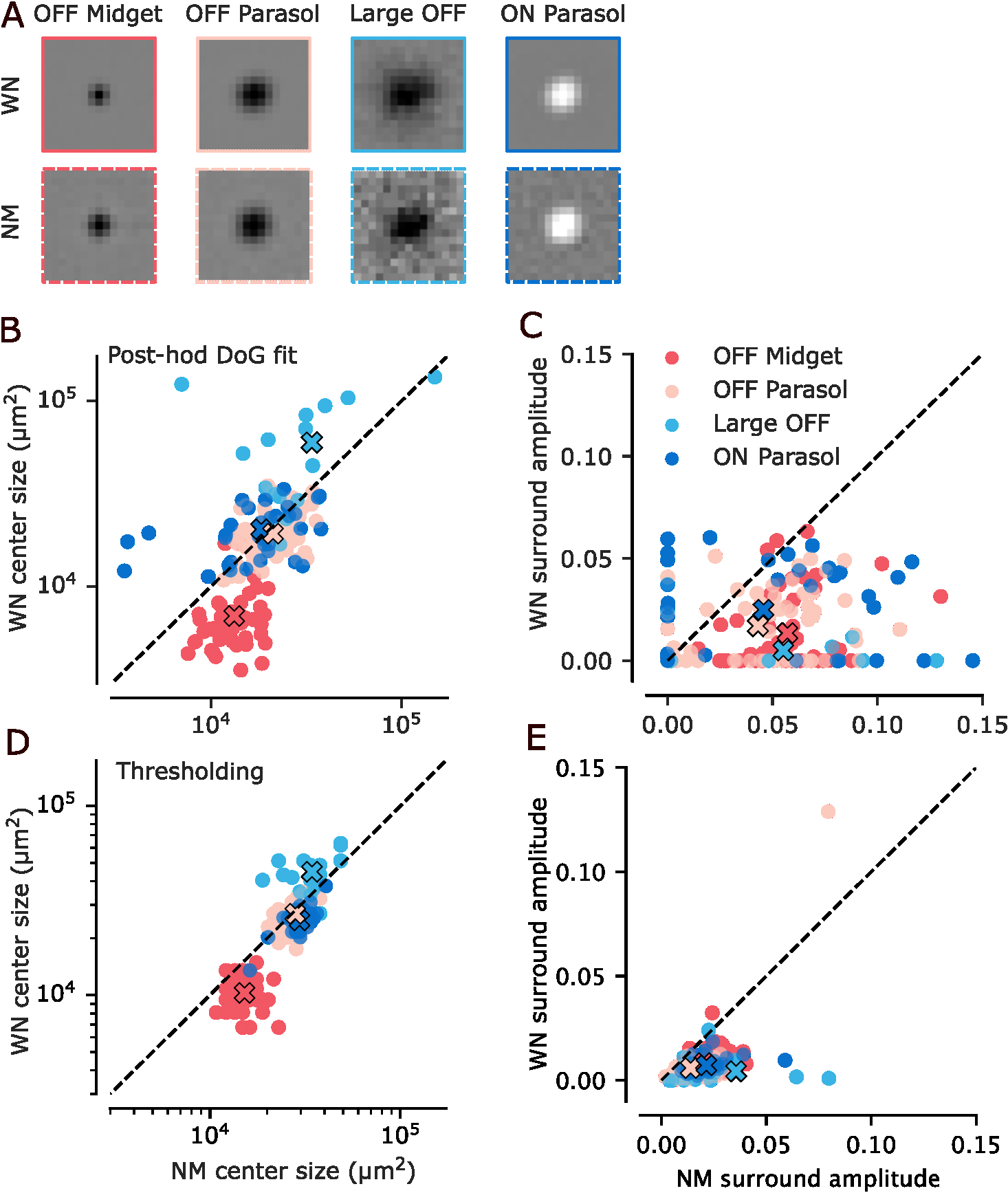

Supplement: Figure 1-1 — A. Cell-type-specific spatial receptive field comparison: Average spatial receptive field across neurons. First row: white noise. Second row: natural movies. B., D. Size of receptive field center for white noise vs. natural movies when fitting a DoG to the spatial filter post-hoc (B) and when using smoothing and thresholding (D). C., E Strength of receptive field surround for white noise vs. natural movies when fitting a DoG to the spatial filter post-hoc (C), and when using smoothing and thresholding (E). Values below 1e−4 clipped to 0. Download Figure 1-1, TIF file. [file eneuro-13-ENEURO.0060-26.2026-s001.tif]
